# Race against death or starvation? COVID-19 and its impact on African populations

**DOI:** 10.1186/s40985-020-00139-0

**Published:** 2020-12-16

**Authors:** Melkamu Dugassa Kassa, Jeanne Martin Grace

**Affiliations:** 1 Discipline of Biokinetics, Exercise & Leisure Sciences, College of Health Science, University of KwaZulu-Natal, 2nd Floor Q Block, Westville Campus, University Road, Durban, 3630 South Africa; 2 Discipline of Biokinetics, Exercise & Podiatric Medicine, College of Health Science, University of KwaZulu-Natal, Westville Campus, University Road, Durban, 3630 South Africa

**Keywords:** Africa, Coronavirus disease, Economic impact, GDP, Political crisis, Social impact

## Abstract

**Background:**

Born in the Chinese city of Wuhan, the consequences of the coronavirus pandemic on global health and economies have been and continue to be devastating. In Africa, its countries grieve for unprecedented burdens of caseloads and mortality due to COVID-19, the virus responsible for the disease. This narrative review aims to establish the scale of the health and economic crisis wrought by the pandemic in Africa, including its impact on the informal economic sector, projections of the effect on national GDP, as well as its political dimensions.

**Methods:**

Documentary evidence issued between January and 8 August 2020 was sought from the Google Scholar, PubMed, Scopus, and Web of Science databases. Searches of published and unpublished abstracts were also conducted from appropriate websites, government documents, organizational reports, newspaper commentaries, and reports issued by global, regional, and local centers of disease control and prevention.

**Results:**

The COVID-19 pandemic is responsible for a fourfold crisis in Africa: (1) a health crisis: the victimization of frontline healthcare workers and the looming caseload and death tolls with 1.039 million (12%) cases being confirmed and over 22,966 (2.4%) deaths as of 8 August 2020. The highest death toll was recorded in Southern Africa of 11,024 (48%) followed by North Africa with 6,989 (29.2%) deaths; (2) a social crisis: with the violation of human rights, the killing of citizens by security forces and increased crime. This, in turn, exacerbates social inequalities, the breakdown of households, instances of social unrest, and general impoverishment; (3) an economic crisis: manifested by a decline in GDP and mass unemployment; (4) a political crisis: implementation of measures that may not be appropriate for Africa, discrimination of refugees and immigrants, evacuation of citizens to their home countries, resulting in distrust of political leaders and postponement of national elections, and mounting cases of conflicts and unrest.

**Conclusion:**

Lockdown during the COVID-19 outbreak is a prevention mechanism in affluent countries, in contrast to developing regions such as Africa, where it is a race against death and starvation. Policymakers must apply novel and locally relevant prevention and management strategies to cope with this growing disaster.

## Background

The rapid and global spread of the coronavirus disease due to COVID-19 poses the greatest public health and economic crisis the world has faced in over a century. It affects all races, genders, and religious groups, regardless of their economic status [1, 2]. The widespread and growing prevalence of the virus throughout the world is associated with the movement of people from its origins—in the Chinese city of Wuhan—to other, previously non-infected areas due to the easy movement of people through work, tourism, trade, and international travel [3, 4]. As the prevalence of the COVID-19 outbreak and its associated morbidity and mortality are dramatically increasing, it poses significant limits on freedom of social life, liberty of travel, social cohesion, and productivity among the global community [5, 6].

Following the spread of the disease across and then outside China, on 3 January 2020, the World Health Organization (WHO) declared the situation a global public health emergency—calling it a pandemic for the first time—and expressed great apprehension about the need to safeguard countries with limited resources, fragile health systems, and lack of preparedness to avert the health crisis that was about to hit them [7, 8]. Taking into consideration the possibility of the auxiliary consequences, the probable effect on human health, the efficiency of present-day readiness, and response measures, the WHO released US$1.8 million from its Contingency Fund for Emergencies (CFE) to support preliminary preparation and response activities [9]. Realizing the growing number of cases in developed countries, and the inability of their health systems to deal with the overwhelming number of sick people who needed critical care, African countries such as Kenya, South Africa, Rwanda, and Uganda started to take preventive measures in early February 2020 that included lockdowns, flight bans, shutting down educational institutions such as schools and universities, closing international borders, banning public transport, and imposing curfews [10, 11].

The outbreak of COVID-19 devastated the networks of African populations, particularly as they affected meetings, tourism, travel, trade, and physical contact due to the territorial and boundary lockdown of countries to limit, and ideally prevent, the spread of the disease. It obliged African communities to keep in touch through advanced technologies, such as the internet, webinars, smartphones, and other electronic systems. Despite the lockdowns in many countries, COVID-19’s prevalence and spread are escalating exponentially, with over 19.8 million cases and over 729,891 deaths at the time of finalizing writing of this article on 8 August 2020 [12]. The numbers that succumbed to the disease in other countries soon surpassed those recorded initially in China, such as the USA, Brazil, India, Russia, South Africa, and Mexico [13]. While the COVID-19 outbreak in China was in its maturation stage, it was at its peak in many developed countries such as, initially, Italy, then France, Germany, Spain, the USA, and the UK through transmission by contagious immigrants and then domestically [14].

The global impact of the disease is massive and overwhelming, causing extensive morbidity, mortality, social disintegration, and economic loss [15]. In Africa, the spread of the outbreak is in its growth stage—initially in Egypt, then Algeria, South Africa, Morocco, Ghana, and Nigeria with their considerable concern over a possible future high transmission rate due to poverty and socio-economic factors [16, 17]. Unlike European and other rich countries, the cultures of African populations are unique in that they appreciate collectiveness and cohesive life, which conflicts with the imposition of lockdowns, curfews, social distancing, and isolation, and no doubt increases the rates of transmission, morbidity, and mortality [18]. Mass gatherings at religious events, large-scale social occasions, traditional funerals, and wedding ceremonies are standard African cultural practices [19]. These, together with a lack of sufficient knowledge and information about the disease, and poor implementation of the information in practice, contribute to the spread of the coronavirus [20]. In contrast, evidence from countries such as China, Korea, Japan, and Turkey confirms that self-care and social isolation could significantly diminish the spread of infection [21]. If temporary cultural reform is not implemented in Africa, the number of cases and the death toll due to the outbreak will surpass the total global cases and deaths [19, 22].

In contrast to conditions in rich countries in the West, the lives of many Africans are based on the daily routine of obtaining enough food to survive [23, 24]. Subjecting African communities to lockdown at home for extended periods is impossible, as people need to access their fields or otherwise find a way to acquire food. For instance, in Ethiopia, Kenya, Nigeria, and South Africa, many have violated the lockdown measures [25–27] and moved through public spaces like restricted areas in towns and market places in large numbers to search for or collect their daily food, despite such moves being forbidden [28]. While lockdown is intended as a measure to reduce the outbreak of COVID-19 and prevent its transmission in the affluent world, it represents a race against death to survive among many in Africa, where life has become an “avoidance–avoidance” conflict situation; be locked in at home and die of starvation, or violate the lockdown regulations to work and collect food and die from the disease. Whatever citizens choose to do, the race is against death, from starvation, or the virus. In Africa, the public perspective about the morbidity and mortality wrought by the disease remains trivial [20], making it especially difficult to implement preventive mechanisms to stop its spread in most countries [29]. This huge obstacle is compounded by social practices: the impact of traditional greetings; poor sanitation (the injunction to wash hands with soap when clean water is scarce) [30]; material sharing, such as of door handles, toilets, television remotes, mobile phone, and computer keyboards; the general lack of caution and egotism that has been predicted to double the risk of coronavirus infections in Africa [31]. Evidence from China showed a marked decline in its transmission after material sharing was banned and lockdown and social distancing were implemented [32], which is impossible in Africa. This is because the majority of Africans live below the poverty line, and millions more are engaged in informal work. In Africa, even if they stay at home, these workers and their families remain exposed to the virus because of overcrowded and unsanitary living conditions that make physical distancing and hygiene nearly impossible. Moreover, most African populations lack access to running water that not only limits the possibilities for handwashing, but also forces women to queue for water, thereby risking themselves and these with whom they come into contact [32].

A community-based and self-care management approach to fight the coronavirus pandemic has been encouraged because of its extensive influence on morbidity and mortality globally, the resulting financial consequences, and the public significance of its prevention [33]. Effective measures against COVID-19 require collaboration among governmental and non-governmental organizations, private agencies, healthcare facilities, and social institutions, such as schools, universities, and religious centers, to mitigate the spread of the virus [34]. If appropriately implemented, such a collaborative effort will enable a healthcare system to cope better with its impact on high-risk individuals and groups as part of the global prevention strategy [35].

Evidence from Africa shows also that the prevalence of the disease is higher among people who travel abroad, gather in large numbers, wash their hands infrequently, have close physical contact with others, and share hand-used materials, all of which increases their opportunities to come into contact with infected people or the virus [36]. Transmission of the coronavirus continues to increase, and its prevalence has spread among all African regions, regardless of their healthcare system and economic level [37, 38]. This has raised the need for further health, social, economic, and political impact analysis studies to assist decision-makers regarding service delivery by indicating the public health requirements that need attention, and by estimating the funding and actions required for appropriate preventative measures. It is, therefore, essential to undertake a wide-ranging analysis when assessing the impact of the COVID-19 outbreak on the health and economic growth of poorly resourced countries, such as those in Africa—such studies not having been reported hitherto. The current evidence focuses mainly on the number of confirmed cases, recoveries, and deaths; the social, economic, and political impacts remain largely unknown. In this narrative review, we aimed to establish the number of COVID-19 cases, recoveries, and deaths; the health crisis’s impact on the formal and informal economic sectors; and projections of gross domestic product (GDP) changes in Africa before, during, and potentially after the pandemic, as well as the political and other consequences of preventative measures such as lockdowns.

## Methods

### Sources of information

We conducted electronic searches of relevant reports, issued between January and 8 August 2020, extracted from the Google Scholar, PubMed Central, Scopus, and Web of Science databases. Also, we searched published and unpublished abstracts, as well as reference lists and tables of contents of relevant journals such as The Lancet, New England Journal of Medicine, and Nature; and examined African-related global and regional government documents, WHO global and regional reports, as well as updates from regional and local centers of disease control and prevention (RLCDC), and also newspaper commentaries from The Guardian (UK) and The Washington Post (USA).

### Search terms in the survey

The following search terms were used: “infectious disease” AND “Corona Virus” AND “Wuhan, China” AND “COVID-19 outbreak” OR “COVID-19 Pandemic” OR “Prevalence of COVID-19”, AND “Serious Acute Respiratory Syndrome (SARS)” AND “impact of COVID-19” OR “economic impact of COVID-19” OR “informal economy” AND “Gross domestic product” OR “GDP” OR “health impact of COVID-19” OR “social impact of COVID-19” OR “political impact of COVID-19” AND “fatality due to COVID-19”, AND “COVID-19 case report”, OR “COVID-19 death reports” AND “social distancing” AND “lockdown”.

### Selection criteria

We two authors independently assessed all titles and abstracts of identified studies and applied the following four inclusion criteria to determine if the reports warranted further investigation: (1) did they include African counties? (2) were they published in English? (3) were the contents pertinent to the aim of the study? (4) were they published between January and July 2020? Full-text versions of journal articles or other source documents were obtained of studies that met the inclusion criteria; for those where this was unclear, we reached consensus after a discussion. From the final selection of source documents, we (J.G. and M.K.) independently abstracted data onto standard data forms. The authors of studies with missing data were contacted by email to obtain the data where possible.

### Estimates of gross domestic product

The pre-pandemic, current, and post-COVID-19 GDP projections for Africa were calculated based on data drawn from the World Economic Outlook database for the continent [39, 40].

### Results of the search for source material

**Fig. 1:**
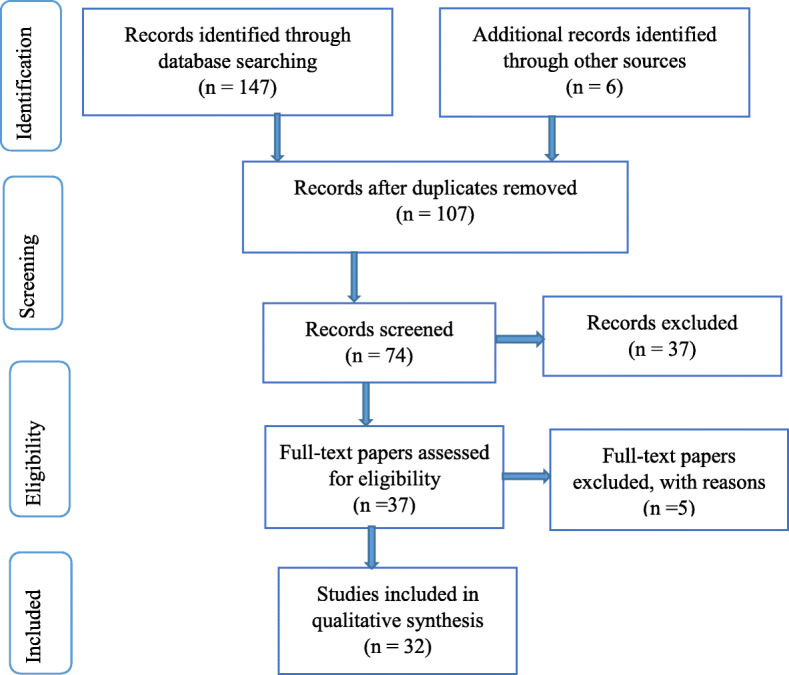
The process of screening and selecting data from the literature for the narrative review

Initially, we identified 136 studies using the search criteria (Fig. 1), using Mendeley Desktop reference management to identify duplication and data management. After removing duplications, checking the eligibility of full-text articles, applying exclusion criteria, and screening, 32 papers met the inclusion criteria.

### The health crisis in Africa

Since the first case of COVID-19 in Africa was confirmed in northern Egypt on 15 February 2020, the pandemic and its associated morbidity and mortality have dramatically increased in the five regions of Africa (North Africa, Central Africa, East Africa, West Africa, and Southern Africa). As the virus reached the continent only 3 months after having first been reported by the WHO in China, and 1 month after it arrived in northern Italy—subsequently and for a period considered the epicenter of the disease—it was to be expected that the number of cases would be lower in Africa. As illustrated in Table 1, as of 8 August 2020, a total of 9.2 million COVID-19 tests had been performed in Africa, with over 1 million cases being confirmed carriers of the virus, over 707,877 people have recovered, and 22,966 deaths being registered.

**Table 1:** COVID-19 tests, confirmed cases, deaths, and active cases in African regions

Region	Tests *N* (%)	Confirmed cases *N* (%)	Recovered *N* (%)	Deaths *N* (%)
North Africa	1,736,200 (20)	171,251 (17)	99,881 (14.5)	6989 (29.3)
Central Africa	327,007 (4)	50,067 (5)	36,454 (5)	952 (4.3)
West Africa	1,193,973 (14)	136,952 (13.5)	106,986 (15.5)	2042 (9)
East Africa	1,685,411 (19.5)	85,801 (8.5)	47,612 (7)	1909 (8.5)
Southern Africa	3,681,823 (42.7)	565,003 (56)	400,065 (58)	11,024 (48)
Total	8,624,414 (100)	1,009,074 (100)	690,998 (100)	22,916 (100)

Source: World meter and Johns Hopkins University as of 8 August 2020 [12, 13]

Southern Africa conducted the highest number of tests at 4 million (43% of the total COVID-19 tests conducted), whereas Central Africa did the least 327,007 (4%). Southern Africa also has the highest number of cases over 565,003 (56%), followed by North and West Africa with 171,251 (17%) and 136,952 (13.6%) cases, respectively. The highest death toll was recorded in Southern Africa at 11,424 (48%), followed by North Africa with 7030 (29.3%) deaths. The countries with the highest number of confirmed cases then were South Africa (553,188), Egypt (95,314), Nigeria (46,140), Ghana (40,533), and Algeria (34,693). The six countries with the highest virus-related registered deaths are South Africa (10,210), Egypt (4992), Algeria (1293), Nigeria (942), Sudan (773), and Morocco (480).

### The dynamic resilience of African states and societies in the face of the COVID-19 pandemic

Current data show that COVID-19 had a greater impact on countries with more “developed” economies than poorer countries on the continent. On the other hand, urban areas had a higher disease burden than rural parts. We reviewed data from The Coronavirus Resource Centre at Johns Hopkins University, displayed in Table 2, which shows that African countries have case fatality rates ranging from 0.5% in Ghana to 8.1% in Chad, compared to 15.0% for the UK, 14.1% for Italy, and 3.3% in Brazil. These relatively low COVID-19 case fatality rates in Africa could be attributed to inefficient social and healthcare data collection systems, although they are more likely due to the youthfulness of African populations [41].

**Table 2:** COVID-19 case fatality rates, comparisons among several countries

Country	Number of cases	Deaths	Case fatality rate %
Sudan	11,894	773	6.5
Nigeria	46,140	942	2.0
Chad	942	76	8.1
Ghana	40,533	206	0.5
Algeria	34,693	1293	3.7
South Africa	553,188	10,210	1.8
Egypt	95,314	4,992	5.2
Kenya	25,837	418	1.6
Ethiopia	22,253	390	1.8
India	2,212,429	44,457	2.3
USA	5,166,319	165,269	3.2
Brazil	3,018,286	100,667	3.3
Italy	250,566	35,205	14.1
UK	310,825	46,574	15.0
France	197,921	30,324	12.9

Source: The Coronavirus Resource Center at Johns Hopkins University 9 August 2020 [41]

### The impact of COVID-19 on the informal economy of Africa and the GDP crisis

As much as 80–90% of economic activities in sub-Saharan Africa derive from the informal sector, which in turn contributes up to 40% to the GDP [42, 43]. The outbreak of the COVID-19 pandemic has profoundly affected this economic sector, which involves informal workers and enterprises [42], in such activities as providing accommodation and food services, manufacturing, wholesale, and the retail trade, including over 500 million farmers who were producing for the urban market. Logistical challenges within supply chains, particularly cross-border and domestic restrictions of movement, may lead to disruptions in the food supply, undermining informal workers’ food security. Also, informal produce markets play an essential role in ensuring food security in many countries, both as a source of food and a place for smallholder farmers to sell their goods. Their closure would lead to increased food insecurity and poverty [44].

The overwhelming majority of workers in the informal economy experience a heightened exposure to occupational health and safety risks due to a lack of appropriate protection and an increased likelihood that they will suffer from illness, accidents, or death. COVID-19 adds to these risks. If they fall sick, most workers, including migrants, do not have guaranteed access to medical care and no income security through sickness or employment injury benefits [45]. If they are unable to access health care, the virus would spread more widely, with fatal consequences. If they can access health care, many will incur out-of-pocket costs that will force them to go into debt or to sell their productive assets, plunging them into deeper poverty [45, 46].

Table 3 presents a comparison of projected changes in the gross domestic products (GDP) of various African states before, currently, and after the pandemic has passed. Table 3 illustrates that the GDP for Africa as a whole is anticipated to shrink from growing at 2.4% before the outbreak to declining by 2.4 to 5.1% during its height; some projections show that it will rise to 4.1% thereafter. The GDP of sub-Saharan Africa and the middle- and low-income countries is reported to change by − 1.6%, − 3%, and 1.6% respectively during the COVID-19, with a projected growth of 4.2%, 4.9%, and 4.9% respectively after the pandemic has passed [35, 47].

**Table 3:** Projected estimates of GDP for various African regions and individual countries before, currently, and post-COVID-19 outbreak [35, 47]

Gross domestic product	Pre (2019) (%)	During (2020) (%)	Post (2021) (%)
Africa	2.4	− 2.4 to − 5.1	4.1
SSA	3.1	− 1.6	4.2
OE	1.7	− 2.9	2.5
Angola	− 1.5	− 1.4	2.6
Chad	3	− 0.2	6.1
Gabon	3.4	− 1.2	3.6
Nigeria	2.2	− 3.4	2.6
DRC	− 0.9	− 2.3	3.4
MIAC	2.3	− 3	4.9
Cameroon	3.7	− 1.2	4.1
Ivory Coast	6.9	2.7	8.7
Ghana	6.1	1.5	5.9
Senegal	5.3	3	5.5
South Africa	0.2	− 5.8	4
Zambia	1.5	− 3.5	2.3
LIAC	5.6	1.6	4.9
DRC	4.4	− 2.2	3.5
Ethiopia	9.0	3.2	4.3
Kenya	5.6	1	6.1
Madagascar	4.8	0.4	5.0
Mali	5.1	1.5	4.1
Tanzania	6.3	2	4.6
Rwanda	10.1	3.5	6.7
Uganda	4.9	3.5	4.3

*SSA* Sub-Saharan Africa, *OE* Oil exporters, *MIAC* Middle-income countries, *LIAC* Low-income countries

The health, social, economic, and political impacts of the COVID-19 pandemic in Africa are outlined in Table 4. The consequences for health are overwhelming, adding to the continent’s already under-resourced health systems and premature deaths due to such underlying conditions as lower respiratory tract infection, HIV/AIDS, diarrheal diseases, malaria, and tuberculosis, which collectively accounted for over 3 million premature deaths in 2016 [47, 48]. In many rich countries, comorbidities complicate the health outcomes of people with COVID-19; in the case of large populations that are undernourished and experience underlying medical conditions, these may put them at increased risk of succumbing to the virus, but this effect is unknown. The poor health service infrastructure on the continent also makes it unlikely that many of those who are infected will be able to receive the care needed at appropriately equipped facilities [24].

**Table 4:** Health, social, economic, and political impacts of COVID-19 in Africa [13, 41, 52–66, 69–85]

Type	Impact
Health impact	1,039,678+ confirmed cases and 22,966+ deaths Increased risk of morbidity and mortality among people with comorbidities, disabilities, and the elderly Victimization of frontline healthcare workers and increased strain on under-resourced health systems Lack of appropriate medical treatment infrastructure Increased burden on existing diseases like HIV/AIDS, tuberculosis, malaria, diarrhea, and lower respiratory tract infections
Social impact	Breakdown in social cohesion following social distancing and interpersonal isolations Majority of academic institution closed Travel bans at international, national, and local levels Food markets closed, limited access to food sources Starvation increased among vulnerable communities Religious gatherings restricted Fear-borne isolation and discrimination created Huma rights violation, growth in illegal practices such as increased crime False news on social media leads communities to incorrect virus prevention responses Exacerbated inequalities, victimization of women and the elderly
Economic impact	Suspension of the aviation and shipping industries for goods and people Primary, secondary, and tertiary industries affected by varying states of lockdown Production of domestic products limited Tourism industry paused and possibly affected long-term Value of supply chains decreased Import and exports diminished, affecting foreign revenue Trade and industry suspended Increased debt to address the pandemic Supply and demand for certain products reduced Financial recession/depression Increase in unemployment with reduced per-capita income Increased pressure on monetary and fiscal policies
Political impact	Governmental, non-governmental, and private sector organizations closed Countries shut their borders, preventing the movement of goods and services Discrimination of refugees and immigrants increased Evacuation of citizens to their home countries result in distrust among leaders Postponement of national elections exacerbate conflicts and unrest Implementation of measures like handwashing may not be appropriate for Africa

The outbreak of the pandemic has greatly affected the social lives of Africans, limiting material sharing, disrupting family lives, and breaking down social cohesion due to social distancing and enforced isolation. Most educational institutions in Africa have been closed. Traditional life in Africa is cohesive and based on mutual dependence that makes social distancing and isolation very difficult. The lockdowns imposed by national governments not only exacerbate existing inequalities but have contributed to the victimization of women with an increase in rape cases [49], of disabled people and the elderly who are the most economically deprived, vulnerable segment of the population [50]. Moreover, produce markets have been closed, limiting people’s access to food sources that have resulted in starvation among vulnerable communities, which were already living below the poverty line (Table 4). In some African countries such as Ethiopia, Kenya, South Africa, and Uganda, lockdowns have resulted in the violation of human rights, the killing of citizens by security forces, and increased traditional crime [28]. This, in turn, has resulted in outbreaks of social unrest, and further impoverishment of communities [51].

Many countries in Africa were already economically compromised by corruption, maladministration, and poor political leadership [39, 47]. Nevertheless, some have shown notable economic growth over the last half-decade, such as Djibouti, Ethiopia, Guinea, Ivory Coast, Senegal, and Tanzania [39, 52]. Many countries have high formal unemployment rates, with people relying on small-scale farming and informal trading to make a living. In those countries that have implemented measures such as lockdowns, which have brought economies to a standstill, many thousands of jobs have been lost, resulting in a growing population who have no reliable source of income [40, 52]. At a national level, some features of the economic impact of COVID-19 prevention measures are the suspension of informal economic sectors such as street vendors and small informal shops in townships called spaza shops [43]. Additional consequences are a reduction in per capita income and the trade-in domestic products, a decline in the value of supply chains, and increased pressure on fiscal policies and debt, all of which can lead to financial recessions or depression [39, 40, 52].

The prominent political impacts of COVID-19 depicted in Table 4 show an increase in discrimination against refugees and immigrants; the closing of national borders, preventing the movement of goods and services; a breakdown in formerly close relations among African nations; and the evacuation of citizens to their home countries resulting in distrust among leaders [53]. Also, the implementation of measures like handwashing may not be appropriate for Africa due to a shortage of running water [53]. Moreover, postponement of national elections has resulted in deadly conflict, distrust, and conflict between political opposition parties and the ruling governments in some African countries such as Ethiopia [54].

## Discussion

In this narrative review, we aimed to establish the number of COVID-19 cases, recoveries, and deaths, their impact on the informal economic sector as well as projections of gross domestic product (GDP) changes in Africa pre-, during, and post-epidemic. It also aimed to determine the health, social, economic, and political crisis of the COVID-19 outbreak in Africa as well as the implications of the lockdowns imposed to stop its spread in African populations.

### Pre-COVID-19 Africa context

The pre-COVID-19 healthcare situation on the continent focused mainly on infectious communicable diseases such as HIV/AIDS, malaria, tuberculosis, cholera, and other health-related issues like maternal and under five-child mortality, and the usual low emphasis on non-communicable diseases (NCDs) [55]. The pre-COVID-19 social factors in Africa are diverse and unifying. For instance, there is a continuous movement of African communities from one region of the continent to the other region legally for visiting, tours, trade, education, meetings, and/or training or through illegal migration by the initiative of human illicit traffic workers. There is also regular mass worshipping in churches, mosques, beaches, and socialization around campfires with some portions of the population visiting nightclubs, music and cinema concerts, mass marketing, social works, wedding, and funeral ceremonies [19]. However, all the above activities changed due to the COVID-19 pandemic as the government of each African country implemented strategies to protect the life, health, economy, and rights of their respective citizens.

Economies pre-COVID-19, specifically regarding informal sectors and formal economies in the African context, are unique. The informal sector, for example, comprises economic activities that circumvent costs and is excluded from the benefits and rights incorporated in laws and administrative rules covering property relationships, commercial licensing, labor contracts, torts, financial credit, and social systems vis-à-vis to the formal economy. Whereas labor laws protect formal economies, COVID-19 exposed the informal sector (the precarious nature of informal work), as evidenced by the absence of contracts or income protection [45].

Last but not the least issue is the pre-COVID-19 political situation of Africa, where 22 countries prepared for national elections [56]. Elections provide avenues for citizens to hold their leaders accountable through either endorsing their legitimacy or replacing them if they have performed abysmally with elections in Africa characterized by fear and panic as it is considered a ‘do-or-die’ affair [57]. With the ongoing COVID-19 pandemic, there are uncertainties as to whether countries will proceed with elections under their respective election calendars. More specifically, there are concerns about how some autocratic leaders may take advantage of this pandemic to prolong their stay by postponing elections to a later date or even indefinitely [57].

### Morbidity, mortality, and recoveries

Although the pandemic arose in China, the COVID-19 outbreak in Africa with its massive morbidity and mortality in African populations added to the existing threat of infectious and chronic diseases they are already predisposed to [58]. At the time of writing this narrative review, the cases and mortality associated with the virus are higher in the North African region, followed by the Western and Southern African regions [59]. A modeling study in Africa supports the finding of our study depicting that by June 30, 2020, nearly 16.3 million people in Africa will contract COVID-19 with the North African region the most affected area by the virus while the East African region will be the least affected. Collective cases on June 30 are projected to range about 2.9 million in Southern Africa, 2.8 million in Western Africa, and 1.2 million in Central Africa [60, 61]. This variation in caseload and mortality might be due to lower levels of socio-economic development and are likely to record less and slower transmissions at the early stages of the pandemic than higher levels of socio-economic development [62–65].

### Health crisis

The results of our narrative review depict a greater health crisis of the COVID-19 outbreak in Africa. This could be ascribed to the already existing under-resourced health care systems and premature deaths, HIV/AIDS, lower respiratory tract infections, diarrheal diseases, malaria, and tuberculosis already accounting for over 3 million deaths in Africa in 2016 [66–68]. While in many developed countries the comorbidities complicate the health outcomes of people with COVID-19, the effect on larger populations who are undernourished and have conditions that may well put them at increased risk of succumbing to the virus is unknown [69]. The limited health service infrastructure also makes it unlikely that many of those who are infected will be able to seek health care at appropriately equipped facilities [25, 70].

### Social crisis

The social crisis posed on African populations is devastating, including false news [71], racism, and discrimination [71, 72] as one of the restraints that block the initial control and management of the COVID-19 pandemic outbreak. This could be ascribed to life in Africa that is cohesive and based on dependence, which makes social distancing and isolation very difficult. The findings of our study indicated that the impact of COVID-19 is very high among vulnerable people, such as women, older people, people with disabilities, and a higher mortality rate among comorbid individuals [5, 73]. Commentary on Israel’s minority group by [74] substantiated the finding of our review stating that older people and people with disabilities and comorbidities are marginalized in all circumstances during the outbreak of COVID-19 in Africa. It could also be due to a lower level of resistance in comorbid and older people than young and non-comorbid individuals with the literature affirming the finding of our review indicating that hypertension, diabetes, cardiovascular disease, kidney disease, and respiratory disease the most prevalent comorbidities [75].

The results of this review showed the closure of food markets, limited access to food sources, which in turn resulted in starvation with many households not being able to access or afford food. Evidence shows that the outbreak of COVID-19 can cause a considerable rise in poverty among vulnerable households, including decreased access to food [26]. This could also be attributed to a loss of income during the lockdown. Investigators corroborate the findings of our review, revealing a disparate understanding and awareness of the African community about the COVID-19 pandemic with the western African countries showing a better level of knowledge about the virus, these could be due to lessons learned from the Ebola outbreak [76].

### Economic crisis

Many countries in Africa have high formal unemployment rates, a reduced per-capita income, and no reliable source of income with people relying on small-scale farming and informal trading to make a living. In this regard, researchers indicate that many countries in Africa are already economically compromised due to corruption, maladministration, and poor political leadership. At the same time, some have shown considerable economic growth over the last half decades, such as Ethiopia, Ivory Coast, Djibouti, Senegal, Guinea, and Tanzania [52, 77].

Despite a few countries showing economic growth, the outbreak of the COVID-19 pandemic poses a looming impact on the already under strained African economy. Researchers corroborated our results, indicating that some features of the economic impact of COVID-19 prevention measures are a decline in the GDP and the value of supply chain and increased pressure on fiscal policies and debt [52, 78]. The results of our review depict a decline in economic growth in Africa of 2.4% pre-COVID-19 in 2019 [39] to between − 2.4 and − 5.1% during the outbreak of the COVID-19 pandemic era (2020) [41, 52]. The lockdown decreased production and increased cost to curve, the pandemic could contribute to this prediction. Supporting the results of our finding, a situational study by the United Nations Economic Commission for Africa predicts economic growth of less than − 3.3% for the African region in 2020 [79]. This could be due to the reduction of wide-ranging export African regions share with the rest of the world than within African regions. Substantiating our finding, a study in Kenya predicts a decline in the five sector’s contributing to the GDP such as agriculture, tourism, construction, infrastructure development, and manufacturing post-COVID-19 [80]. Moreover, the findings of our review reflected the expectation of a variation of decline in GDP change across African regions before and during the COVID-19 pandemic with a greater GDP loss (− 3%) observed in the middle-income countries, followed by the whole of Sub-Saharan Africa (− 1.6%) during the outbreak of the COVID-19 pandemic [39, 40, 52]. The disparity could be the result of the different states of lockdown and the differences these African regions have in the levels of socio-economic and trade partnership with Europe, China, and America [60, 61]. A study by the World Banking Group depicted that the majority of countries in the African region will show a decline in GDP, which was 2.4% pre-COVID-19, and reduced to − 2.4% during the outbreak of the COVID-19 pandemic period [52, 81]. The decline of GDP is likely to be the result of decreased import and export, job losses due to the lockdown, reduced production of export commodities, and increased debt to address the pandemic [82].

### Political crisis

The political impact of COVID-19 is overwhelming as a result of the existing fragile and weak government structure in addition to the stated health, social, and economic impacts [77]. The findings of our narrative review revealed a breakdown in the connection among African nations and the closing of national borders, evacuation of citizens to their home countries that result in mistrust between frontrunners, and execution of actions that may not be suitable for Africa [51, 83]. In this regard, during the initial outbreak of the COVID-19 pandemic, political decisions taken by African leaders were encouraging, possibly due to fear of their under-strained health system, limited quality health facilities, and an under-strained economy [84]. Also, despite African countries’ public health emergency system that is constrained, the action taken by African leaders to curve the spread of the COVID-19 outbreak is not only faster but also highly acknowledged; they declared a public health emergency and banned irrelevant public movement, followed by lockdown to minimize the possibility of the disease outbreak in their respective countries [53, 84]. Aligned with the differences in economies, health systems, and lifestyles among western and African populations, the health outcome, social impact, and economic toll in both developed and developing countries remain disparate [85]. This might be the result of the lack of social support, far more people living on or below the poverty line, and the fact that more people are self-employed or not employed in the formal sector in African regions.

### Implications

Most African countries’ health care facilities and resources are under stress. If the spread of the pandemic endures for as long as it is predicted, the secondary effects of the COVID-19 pandemic will swiftly become an economic emergency, leading the world towards a recession, severely impacting African countries with limited resources and the most impoverished populations. Moreover, many African countries have implemented WHO-developed COVID-19 management strategies and approaches to deal with the pandemic, such as “quarantine, social distancing, self-isolation, usage of water, sanitation, and wash hands practices.” However, while these approaches to curb and manage the COVID-19 pandemic outbreak are deemed appropriate and successful in Asian countries such as China and South Korea, the strategies remain challenging, impossible, and unacceptable in African countries due to inequalities, substantial job losses, disparity, and informal economic practices in many African countries. Besides, the lockdown is an obstacle to peace, security, and safety to African countries that are plagued by unrest exhibiting illegal practices such as crime, corruption, and maladministration prolonging the existing poverty, starvation, and inequality.

## Conclusion

The COVID-19 pandemic has impacted and will continue to have a long-term impact on the organization of our health systems, of our production, processing and exchange of goods and services, as well as our consumption practices, cultures, and our relationship with life and death. The study we have just evaluated contributes to understanding these changes and their impact. It is the choice of policymakers and other stakeholders to consider these findings in the prevention and response to new epidemics. To successfully address the health, social, economic, and political impact of the COVID-19 pandemic outbreak, a united, active, and well-organized public health emergency reaction is required among leaders, governmental and non-governmental organizations. The success of public health emergency readiness and response is substantially based on the superiority and volume of information accessible during the outbreak of the pandemic. Rapid communiqué and distribution of wrathful information are required to prevent and manage the health, social, economic, and political impact of the COVID-19 pandemic and to ensure it is a race neither against death nor against starvation for African populations. Therefore, African countries require culturally relevant and indigenous pandemic managing strategies in years to come to make them responsive to pandemics that could affect their economies, and citizens’ health and lives.

## Data Availability

All information incorporated in this narrative review can be accessed from both the corresponding author and the second author on a timely appeal.

## Abbreviations

BREC: Biomedical Ethical Review Committee
CFE: Contingency Fund for Emergencies
COVID-19: Coronavirus 19
GDP: Growth domestic product
LIAC: Low-income African countries
MIAC: Middle-income African countries
OE: Oil exporters
RLCDC: Regional and Local Centers of Disease Control and Prevention
SSA: Sub-Saharan Africa
UK: United Kingdom
UKZN: University of KwaZulu-Natal
USA: United States of America
WHO: World Health Organization
